# The Role of Aldosterone in Detecting Resistance-Driven Hypoaldosteronism and Deficit-Driven Hypoaldosteronism

**DOI:** 10.3390/jcm15010218

**Published:** 2025-12-27

**Authors:** Jorge Gabriel Ruiz-Sánchez, Alfonso Luis Calle-Pascual, Miguel Ángel Rubio-Herrera, Paz De Miguel Novoa, Emilia Gómez-Hoyos, Isabelle Runkle

**Affiliations:** 1 Servicio de Endocrinología y Nutrición, Instituto de Investigación Sanitaria Fundación Jiménez Díaz (IIS-FJD, UAM), Hospital Universitario Fundación Jiménez Díaz, 28040 Madrid, Spain; 2 Facultad de Medicina, Universidad Complutense de Madrid, 28040 Madrid, Spain; 3 Servicio de Endocrinología y Nutrición, Instituto de Investigación Sanitaria del Hospital Clínico San Carlos (IdISSC), Hospital Clínico San Carlos, 28040 Madrid, Spain; 4 Centro de Investigación Biomédica en Red de Diabetes y Enfermedades Metabólicas Asociadas (CIBERDEM), 28029 Madrid, Spain; 5 Servicio de Endocrinología y Nutrición, Hospital Clínico Universitario de Valladolid, 47003 Valladolid, Spain

**Keywords:** hypoaldosteronism, isolated hypoaldosteronism, aldosterone, hyperreninemia, hyperkalemia

## Abstract

**Background/Objectives**: Hypoaldosteronism is classified into “aldosterone deficit” (Aldo-D) and “aldosterone/mineralocorticoid resistance” (Aldo-R) based on etiopathogenic mechanisms. This distinction could be useful for guiding the treatment. However, no reliable methods have been established to differentiate these subtypes. We first aimed to assess whether aldosterone levels could help identify them when assessed in the setting of hyperkalemia or hyperreninemia. **Methods**: We conducted a retrospective analysis of eighty-four cases of hypoaldosteronism. Aldo-D and Aldo-R classification was based on the presence of clinical factors associated with aldosterone deficit and mineralocorticoid resistance, respectively. The accuracy of plasma aldosterone (PAC) to identify each type of hypoaldosteronism individually was evaluated using AUC-ROC analysis. **Results**: Aldo-D was identified in 66 (78.6%), and Aldo-R in 41 (48.8%) cases. Factors related to both subtypes were observed in forty-seven (56%) cases. AUC-ROC analysis of PAC measured during hyperkalemia showed low accuracy for detecting either subtype. During hyperreninemia, PAC accuracy was adequate for identifying Aldo-D but unsatisfactory for Aldo-R. Nevertheless, a PAC ≤ 60 pg/mL (6 ng/dL, ~166 pmol/L) during hyperkalemia and hyperreninemia yielded positive predictive values (PPV) of 94% and 100%, respectively, for Aldo-D, while a PAC value > 160 pg/mL (~443 pmol/L), particularly ≥ 200 pg/mL (20 ng/dL, ~550 pmol/L) in either condition had a PPV of 100% for Aldo-R. **Conclusions**: Although overall diagnostic accuracy was limited, extreme low and high PAC values (≤ 60 pg/mL or ≥ 200 pg/mL) may be suggestive of Aldo-D or Aldo-R, respectively, while intermediate values remain difficult to interpret due to substantial overlap.

## 1. Introduction

Aldosterone, the most important mineralocorticoid in humans, is synthesized in the adrenal gland’s zona glomerulosa. Its production is primarily regulated by the following two stimuli: angiotensin-II (via the renin–angiotensin–aldosterone system RAAS) and the blood potassium concentration ([K+]) [1]. Elevations in renin, and consequently, angiotensin-II (AngII) levels lead to an increase in adrenal aldosterone secretion. Additionally, a decrease in the former leads to a descent in the latter. Elevated [K+] (hyperkalemia) enhances aldosterone production, whereas low [K+] (hypokalemia) suppresses it. Furthermore, adrenocorticotropic hormone (ACTH) also plays a role in aldosterone production [2], particularly in its circadian rhythm and acute regulation.

Aldosterone is crucial for sodium (Na+) and potassium (K+) balance as well as volemic and acid-base homeostasis. Acting on mineralocorticoid receptors in the distal convoluted tubule and the proximal collecting duct of the nephron, it promotes Na+ reabsorption into the bloodstream and K+ urine excretion. Additionally, its action on intercalated cells facilitates urinary hydrogen ion excretion.

Hypoaldosteronism is produced by a deficiency or impaired action of aldosterone at the distal nephron and is characterized by impaired renal potassium and hydrogen-ion excretion, as well as reduced distal tubular sodium reabsorption [3,4]. As a result, these defects lead to the development of hyperkalemia, which can be severe. The associated natriuresis can also reduce circulating effective volume, predisposing individuals to hypovolemic hyponatremia with inappropriate urinary sodium losses [5]. As a consequence, the typical biochemical consequences of hypoaldosteronism include elevated serum potassium levels and hypovolemic hyponatremia, accompanied by hyperchloremic metabolic acidosis in closely half of cases [4,6]. Thus, this endocrinological syndrome encompasses a broader clinical spectrum that extends beyond hormone levels.

Most cases of acquired hypoaldosteronism occur in individuals exposed to medications or disorders that interfere with aldosterone production or action, or damage the distal tubular epithelium [4]. These factors can be grouped into two mechanistic categories as follows: (1) those that reduce circulating mineralocorticoid levels, and (2) those that impair mineralocorticoid action—either through pharmacologic antagonism of the mineralocorticoid receptor, blockade of epithelial sodium channels, or structural injuries that compromise distal tubular responsiveness. Thus, hypoaldosteronism can be clinically classified into “aldosterone deficit” (Aldo-D) or “aldosterone/mineralocorticoid resistance” (Aldo-R)—the latter also referred to as pseudohypoaldosteronism—as a function of the presence of any of these factors [3]. Importantly, many patients may exhibit a combination of reduced aldosterone levels and impaired distal tubular responsiveness, resulting in mixed mechanisms of hypoaldosteronism.

Some authors have postulated that the classification of hypoaldosteronism into Aldo-D and Aldo-R groups could be useful for selecting appropriate therapy [7,8], and therefore may be clinically relevant. Unfortunately, diagnostic strategies for hypoaldosteronism remain poorly defined [9], with no established methods for distinguishing its subtypes.

We hypothesize that determining whether aldosterone is appropriately elevated, suppressed, or inappropriately normal—while simultaneously assessing its response to stimulatory factors such as hyperkalemia or hyperreninemia (an indirect marker of AngII and hypovolemia)—could clarify the underlying mechanism. To address this, we aimed to evaluate whether aldosterone levels assessed during hyperkalemia and/or hyperreninemia could be of use in the differential diagnosis of hypoaldosteronism subtypes.

## 2. Materials and Methods

This is a retrospective study of adult patients with hypoaldosteronism assessed by the Endocrinology Department of the Hospital Clínico San Carlos of Madrid, Spain, from January 2012 to August 2019. The complete methodology of the registry has already been reported [4]. Briefly, the original registry contains the data of 112 hypoaldosteronism cases, of which 106/112 (94.6%) had hyperkalemia, 61/112 (54.5%) hypovolemic hyponatremia, and 60.3% metabolic acidosis (35 of the 58 cases in which acid-base status was evaluated), all of them with inadequate K+ excretion [10].

In the current study, we selected only those cases which had concomitant aldosterone and blood ion measurements (n = 84). Patients with hypoaldosteronism following adrenal surgery or secondary to excessive doses of eplerenone or spironolactone in the therapy of primary aldosteronism were excluded from the registry, as were patients with hypervolemia (heart failure, cirrhosis, and third space) or oliguric renal failure. The study was performed according to the standards of good clinical practice and Helsinki Declaration and approved by the local ethical committee of the Hospital Clínico San Carlos (Cod. 20/714-E_BS, 14 December 2020). Written informed consent was waived.

### 2.1. Collected Data and Definition of Variables

The following variables were collected from clinical records: a history of hypertension, diabetes mellitus (DM), chronic kidney disease (CKD), obstructive uropathy, renal transplant, urinary tract infection, hypoalbuminemia (albumin ≤ 3.5 g/L), self-reported low-salt diet, prior use of heparin, trimethoprim, cyclosporine, tacrolimus, non-steroidal anti-inflammatory drugs (NSAIDs), β-blockers (βB), aliskiren, angiotensin-converting enzyme inhibitors (ACEIs) or angiotensin 2 receptor blockers (ARBs), mineralocorticoid receptor blockers (MRBs), loop diuretics, thiazides, thiazide-amiloride diuretics, and concomitant glucocorticoid therapy (prednisone, prednisolone, or dexamethasone). The following variables regarding comorbidities and previous treatment were categorized as mineralocorticoid-resistance factors (ResFs) based on prior studies: renal transplant [11,12,13,14,15], obstructive uropathy [16,17,18], urinary tract infection [18,19,20], use of trimethoprim [21,22,23], cyclosporine [15,24], tacrolimus [12], amiloride, and MRB. Furthermore, the following variables were classified as aldosterone-deficit factors (AldDefFs): primary adrenal insufficiency, use of heparin, NSAID, ACEI/ARB, βB, chronic glucocorticotherapy (more than 6 weeks), and aliskiren.

Biochemical and clinical variables for the current study were collected from the moment of aldosterone measurement. These variables were plasma aldosterone (PAC) and direct plasma renin concentration (DRC), both measured by radioimmunoassay in hospital central lab, glycemia, serum creatinine, blood gasometer values, blood and urine ions and osmolality, urine creatinine, and calculated trans-tubular potassium gradient. As per the routine clinical protocol, blood and urine samples are collected early in the morning, while fasting, before 09:00 a.m., and are processed by the laboratory the same morning. All biochemical variables used in the analysis were from morning-processed samples. PAC and DRC were measured after a period of ambulation, followed by at least 5 min in the seated position.

Since no consensus exists regarding cutoff values for defining hyperreninemia in the medical literature, we used the 4th quartile of DRC in the entire cohort to define this variable, as commonly done in studies addressing hyperreninemia that select the highest population-derived thresholds for this purpose [25,26,27]. Likewise, in the absence of an established cutoff to determine a normal aldosterone response to hyperkalemia or hyperreninemia, we applied the PAC ≥ 200 pg/mL (20 ng/dL) threshold based on the study by Dluhy et al. [28] for comparative purposes when applicable. Similarly, the PAC ≤ 60 pg/mL threshold—considered a normal low value based on studies of saline overload [29,30] and used as a defining cutoff of severity of the opposite condition, primary aldosteronism [31]—was also applied in comparative analyses.

Hypoaldosteronism was diagnosed according to the criteria established in Table 1, representing the broad spectrum of this condition and encompassing probable cases of both Aldo-D and Aldo-R. For classification purposes, cases were defined as Aldo-R when at least one ResF was identified. Those without any ResF were categorized as non-Aldo-R. Similarly, Aldo-D was defined when at least one AldDefF was present, whereas the absence of such factors classified the case as non-Aldo-D. PAC levels were not used for hypoaldosteronism classification.

Hyperkalemia was defined as [K+] ≥ 5 mmo/L. Hyponatremia was defined as a serum sodium ([Na+]) ≤ 135 mmol/L after correction for glycemia [32]. Hypovolemia was defined as the presence of the maximum height of the internal jugular pulse (HIJP) below the sternal angle with the patient reclined at 0–30°, in addition to at least two of the following data: thirst, orthostatic symptoms/signs, blood pressure ≤ 90/60 mmHg, heart rate ≥ 90 bpm, decreased eye tone on palpation, distal venous filling of the upper limbs below the diaphragmatic line in a sitting position, and a rise in serum creatinine (SC) accompanying the descent in [Na+] [33,34]. If HIJP was not measured, hypovolemia was determined by the presence of at least three of the other signs/symptoms described above. Patients without symptoms/signs of hypovolemia and with a HIJP at 1–3 cm above the sternal angle were classified as euvolemic.

### 2.2. Statistical Analysis

Categorical variables are given as frequencies and percentages. Quantitative variables are described as mean/medians and standard deviation/interquartile range in accordance with their normality distribution. Chi-squared and Fisher’s tests were used for comparative analysis between categorical variables. T-student/ANOVA test, if parametric, or Mann–Whitney U/Kruskal–Wallis test were used for comparative analyses of parametric or non-parametric variables, respectively. Correlations (r value) with Spearman test for non-parametric or Pearson test for parametric were executed for quantitative variables. The diagnostic accuracy of PAC for detecting hypoaldosteronism cases due to Aldo-D or Aldo-R, according to our classification, was evaluated using the area under the receiver operating characteristic (ROC) curve (AUC) with 95% confidence intervals (95% CIs). Sensitivity, specificity, positive predictive value (PPV), and negative predictive value (NPV) were calculated based on 2 × 2 contingency tables. Youden’s Index was applied to determine the optimal cutoff values derived from ROC analysis. Multivariable linear regression analyses were conducted using the enter (forced-entry) method to identify determinants of PAC. Regression coefficients (B) with 95% CI were reported. Three sequential models were evaluated as follows: Model 1 included DRC, [K^+^], serum creatinine, and [Na^+^]; Model 2 additionally included sex and age; and Model 3 further incorporated ResF and AldDefF. A hierarchical (block-wise) approach was also applied, with Block 1 including age and sex, Block 2 including DRC, [K^+^], serum creatinine, and [Na^+^], and Block 3 including ResF and AldDefF. Changes in R^2^ were used to assess improvements in model fit. Statistical significance was considered when *p* value was <0.05 in two-tailed analysis. The analysis was performed with SPSS version 25 (IBM Corp., New York, NY, USA).

## 3. Results

Of the 84 selected cases with PAC measurements, 39 (46.4%) were women. The median age was 77 years [65–84]. Primary adrenal insufficiency was diagnosed only in four (5.6%) cases; the remaining cases corresponded to isolated hypoaldosteronism.

In 41 (48.8%) of the 84 cases at least one ResF was present (obstructive uropathy: 21, renal transplant: 4, urinary tract infection: 9, use of trimethoprim: 13, cyclosporine: 1, tacrolimus: 4, and MRB: 8), and they were classified as Aldo-R, while the remaining were classified as non-Aldo-R. The clinical characteristics of those cases compared to non-Aldo-R are described in Table 2.

On the other hand, 66 of the 84 cases (78.6%) had at least one AldDefF (primary adrenal insufficiency: 4, use of heparin: 24, NSAID: 8, ACEI/ARB: 43, β-Blocker: 14, aliskiren: 0, and chronic glucocorticotherapy: 10). All of them were classified as Aldo-D, and the rest of cases as non-Aldo-D. The clinical characteristics of Aldo-D cases compared to non-Aldo-D are described in Table 3.

In the total cohort, the combination of both factors (CombinedF) was observed in forty-seven cases (56%). Notably, both cases with Aldo-D and Aldo-R also had ResF and AldDefF, respectively, as can be observed in Table 2 and Table 3.

In the entire cohort, multivariable regression analyses using forced-entry method consistently showed that DRC was the only variable significantly associated with PAC (B = 0.4, 95% CI: 0.3 to 0.5; *p* < 0.001) across all three models. In addition, in Model 3, the number of AldDefF also directly contributed to PAC values (B = −35, 95% CI: −70 to −0.5; *p* = 0.047), with none of the other variables studied showing a significant impact. Hierarchical regression analyses revealed that age and sex (Block 1) did not contribute significantly to PAC. However, the addition of DRC, [K^+^], serum creatinine, and [Na^+^] (Block 2) substantially improved model fit (R^2^ = 0.535), with DRC remaining the sole significant predictor (B: 0.4, 95%CI: 0.3 to 0.5; *p* < 0.001). The further inclusion of ResF and AldDefF (Block 3) led to a modest increase in the explained variance (R^2^ = 0.559), with only AldDefF reaching statistical significance (B = −35, 95% CI: −70 to −0.5; *p* = 0.047) along DRC (B: 0.4, 95% CI: 0.3 to 0.5; *p* < 0.001).

### 3.1. Reliability of PAC Measurement During Hyperkalemia

Hyperkalemia was observed at the time of PAC measurement in fifty-one cases (60%). The presence of hyperkalemia did not significantly affect median PAC values in the entire cohort (82 vs. 95 pg/mL; *p* = 0.372), nor when analyzed separately in the non-Aldo-R and Aldo-R groups, nor in the non-Aldo-D and Aldo-D groups.

No correlation between PAC and [K^+^] was found in the total cohort, neither when analyzing all cases regardless of hyperkalemia status (r Spearman = 0.018, *p* = 0.871) nor when restricting the analysis to cases with hyperkalemia (r Spearman = −0.04, *p* = 0.783).

#### 3.1.1. Accuracy in Identifying Aldo-R During Hyperkalemia

**Figure 1:**
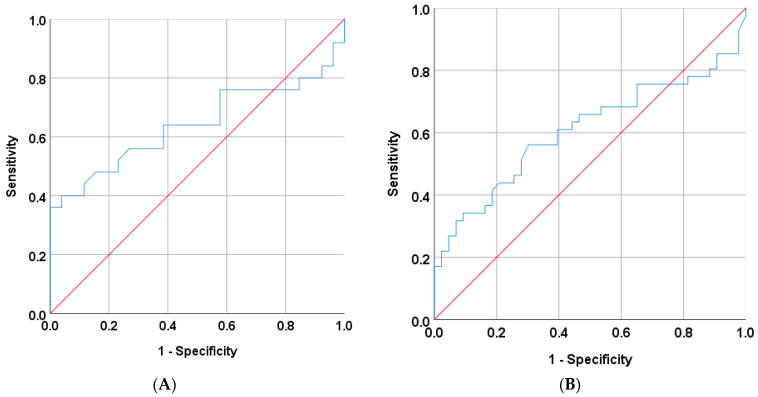
ROC curve for PAC measured during hyperkalemia (**A**) and independently on it (**B**), to identify mineralocorticoid resistance hypoaldosteronism. PAC: plasma aldosterone.

In 51 cases with hyperkalemia, 25 were classified as Aldo-R and 26 as non-Aldo-R. The AUC of PAC for distinguishing Aldo-R from non-Aldo-R was 0.64 (95%CI: 0.5 to 0.8; *p* = 0.083) (Figure 1A). When analyzed in the entire cohort, regardless of the presence of hyperkalemia, the AUC was 0.61 (95%CI: 0.48–0.73; *p* = 0.091) (Figure 1B).

The highest Youden’s Index (0.36) was found at both PAC values of 168 pg/mL and 200 pg/mL. The former cutoff yielded sensitivity of 40%, specificity of 96.2%, PPV of 90.9%, and NPV of 62.5%. Similarly, PAC cutoff ≥ 200 pg/mL achieved sensitivity of 36%, specificity of 100%, PPV of 100%, and NPV of 61.9%, for identifying Aldo-R cases during hyperkalemia.

When the PAC cutoff ≥ 200 pg/mL was assessed regardless of hyperkalemia, it yielded a PPV of 81.3%, NPV of 58.8%, sensitivity of 31.7%, and specificity of 93%.

When those cases presenting CombinedF were excluded, 4 cases remained identified as Aldo-R and 20 as non-Aldo-R. The ROC analysis did not show improvement (AUC: 0.675, 95% CI: 0.29–1.00; *p* = 0.278)

#### 3.1.2. Accuracy in Identifying Aldo-D

**Figure 2:**
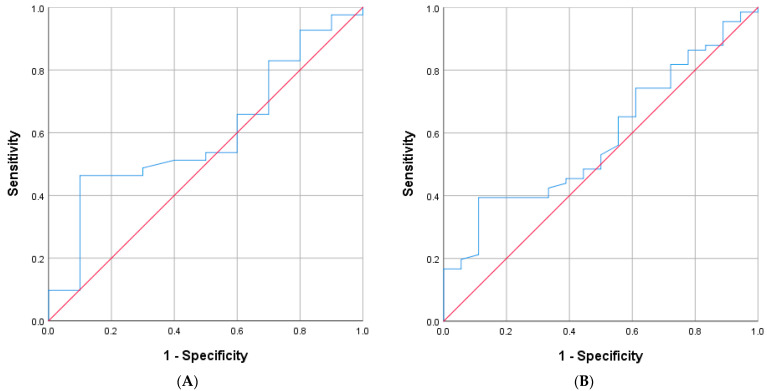
ROC curve for PAC measured during hyperkalemia (**A**) and regardless of its presence (**B**), to identify aldosterone deficit hypoaldosteronism. PAC: plasma aldosterone.

During hyperkalemia, 41 cases were classified as Aldo-D and 10 as non-Aldo-D. The AUC of PAC for detecting Aldo-D was 0.596 (95% CI: 0.41–0.78, *p* = 0.349) when measured during hyperkalemia (Figure 2A), and 0.583 (95% CI: 0.45–0.72, *p* = 0.280) when measured regardless of its presence (Figure 2B).

The highest Youden’s Index was 0.36 at PAC of 63.5 pg/mL assessed during hyperkalemia. This cutoff yielded sensitivity of 46.3%, specificity of 90%, PPV of 95%, and NPV of 29% for identifying Aldo-D cases. Similarly, PAC cut-off ≤ 60 pg/mL showed PPV of 94.4%, NPV of 27.3%, sensitivity of 41.5%, and specificity of 90%.

When evaluated regardless of hyperkalemia, the PAC cut-off ≤ 60 pg/mL yielded a PPV of 92.3%, NPV of 27.6%, sensitivity of 36.4%, and specificity of 88.9%.

When those cases presenting CombinedF were excluded, 10 hyperkalemic cases were identified as Aldo-D and 14 as non-Aldo-D. The ROC analysis showed a trend toward an improvement in diagnostic performance (AUC: 0.718, 95% CI: 0.50–0.93; *p* = 0.074).

### 3.2. Reliability of PAC Measurement During Hyperreninemia

In the entire cohort, PAC and DRC values were strongly correlated (r: 0.704, *p* < 0.001). This relationship persisted only when the analysis was restricted to cases coinciding with hyperkalemia (r: 0.759, *p* < 0.001) but not without it. Within this hyperkalemic subgroup, the correlation remained significant and strong in Aldo-R cases (r: 0.821, *p* < 0.001) but was no longer significant in non-Aldo-R cases (r: 0.283, *p* = 0.162). In contrast, when stratifying by Aldo-D status, the correlation was consistently observed in both Aldo-D (r: 0.83, *p* < 0.001) and non-Aldo-D cases (r: 0.939, *p* < 0.001).

The 4th quartile of DRC in the entire cohort was composed of values above 35 pg/mL, which was used to define hyperreninemia. This range of DRC was observed in 23/84 (27.4%) cases, with significantly higher rates in Aldo-R compared to non-Aldo-R group (39% vs. 16.3%; *p* = 0.019), and not significantly in Aldo-D than non-Aldo-D (30.3% vs. 16.7%; *p* = 0.373).

In the overall cohort, PAC and DRC were strongly correlated during hyperreninemia (r: 0.759, *p* < 0.001), but not in its absence. Furthermore, a trend toward higher median PAC values was observed in those with hyperreninemia than without it (125 vs. 82 pg/mL; *p* = 0.067). This trend persisted within the Aldo-R group (141 vs. 91 pg/mL; *p* = 0.084), but not in non-Aldo-R (83 vs. 77 pg/mL; *p* = 0.936). Similarly, no significant differences were observed in Aldo-D (108 vs. 82 pg/mL; *p* = 0.227) or non-Aldo-D (317 vs. 82 pg/mL, *p* = 0.206), although only three hyperreninemic cases were present in the latter subgroup.

**Figure 3:**
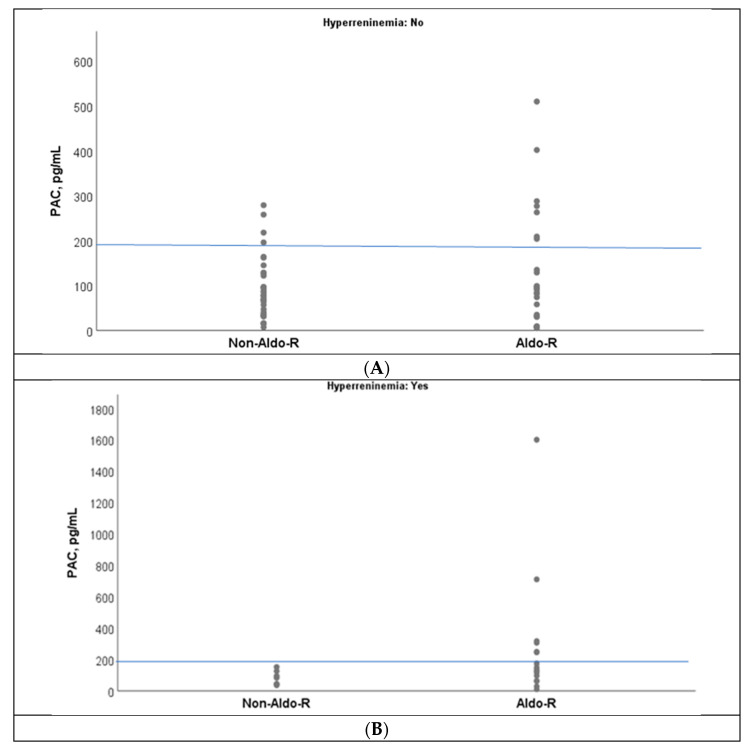
Dispersion of PAC values grouped by non-Aldo-R and Aldo-R, in accordance with the absence (**A**) and presence (**B**) of hyperreninemia. PAC: plasma aldosterone; Aldo-R: aldosterone/mineralocorticoid resistance. The blue line represents the 200 pg/mL cutoff on the Y-axis in both panels (**A**,**B**).

An overlap of PAC values was observed between Aldo-R and non-Aldo-R groups when PAC was below 200 pg/mL, which was indistinct from the presence of hyperreninemia(Figure 3A,B). However, no case with non-Aldo-R had PAC values ≥ 200 pg/mL during hyperreninemia (Figure 3B).

**Figure 4:**
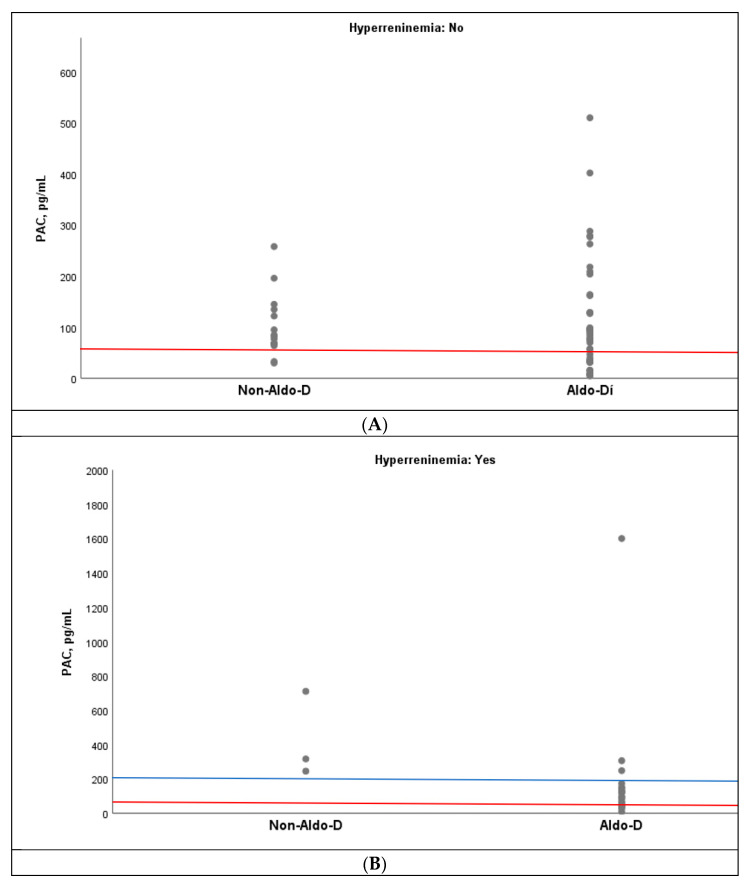
Dispersion of PAC values grouped by non-Aldo-D and Aldo-D, in accordance with the absence (**A**) and presence (**B**) of hyperreninemia. PAC: plasma aldosterone; Aldo-D: aldosterone deficit. The red line represents the 60 pg/mL cutoff in both panels (**A**,**B**) and, the blue line represents the 200 pg/mL cutoff in (**B**).

Similarly, an overlap of PAC values was observed between the Aldo-D and non-Aldo-D groups, both in the presence and absence of hyperreninemia. However, when the cutoff point of ≤60 pg/mL was set, only one case of non-Aldo-D was observed in the absence of hyperreninemia, but none in its presence (Figure 4A,B). In fact, in the latter setting, all three cases with non-Aldo-D had PAC values above 200 pg/mL (Figure 4B).

#### 3.2.1. Accuracy in Identifying Aldo-R

**Figure 5:**
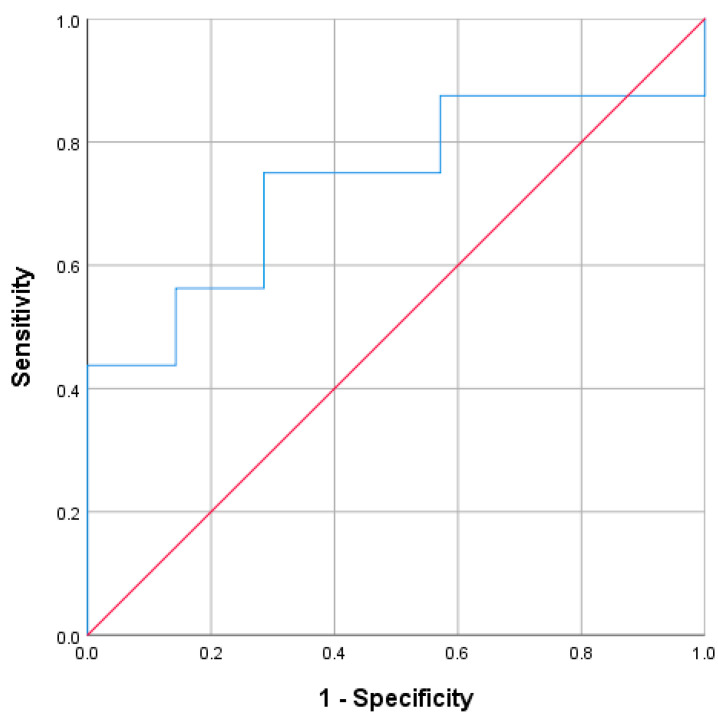
ROC curve for PAC measured during hyperreninemia, detecting Aldo-R (PAC: plasma aldosterone; Aldo-R: aldosterone/mineralocorticoid resistance).

In the 23 cases with hyperreninemia, 16 were classified as Aldo-R and 7 as non-Aldo-R. The ROC analysis showed an AUC of 0.732 (95% CI: 0.53–0.94; *p* = 0.082) for PAC measured during hyperreninemia to differentiate Aldo-R from non-Aldo-R (Figure 5). Based on Youden’s Index, two PAC values showed the highest accuracy. The first, 96 pg/mL (Youden’s Index = 0.46), yielded sensitivity of 75%, specificity of 71.4%, PPV of 85.7%, and NPV of 55.4%. The second, 163 pg/mL (Youden’s Index = 0.44), showed sensitivity of 43.8%, specificity of 100%, PPV of 100%, and NPV of 43.8%. Similarly, PAC value ≥ 200 pg/mL yielded PPV of 100%, NPV of 41.2%, sensitivity of 37.5%, and specificity of 100%.

When those cases presenting CombinedF were excluded, only 9 cases remained available for analysis, 3 categorized as Aldo-R and 6 as non-Aldo-R. The AUC of ROC analysis was 1.00 (95% CI: 1.00–1.00; *p* = 0.002).

#### 3.2.2. Accuracy in Identifying Aldo-D

**Figure 6:**
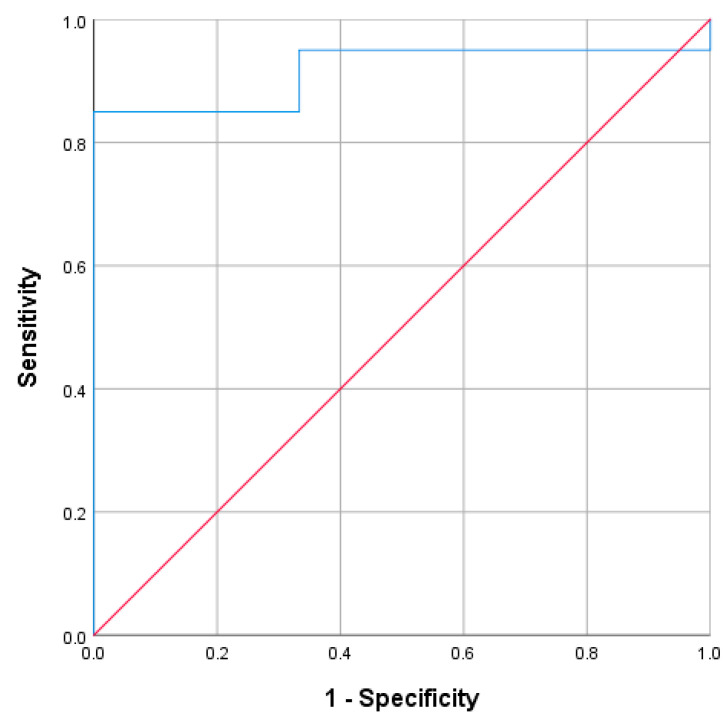
ROC curve for PAC measured during hyperreninemia, detecting Aldo-D (PAC: plasma aldosterone; Aldo-D: aldosterone deficit).

In the 23 cases with hyperreninemia, 20 were classified as Aldo-D and 3 as non-Aldo-D. In order to differentiate non-Aldo-D from Aldo-D, the AUC of PAC measured during hyperreninemia was 0.917 (95%CI: 0.796 to 1; *p* = 0.022) (Figure 6). The PAC value of 210 pg/mL yielded the highest Youden’s Index (0.85), with sensitivity of 85%, specificity of 100%, PPV of 100%, and NPV of 50%. In this context, all five cases with PAC ≤ 60 pg/mL corresponded to Aldo-D, representing sensitivity of 25%, specificity of 100%, PPV of 100%, and NPV of 16.7%.

When those cases presenting CombinedF were excluded, again only 9 cases remained available for analysis, 6 categorized as Aldo-D and 3 as non-Aldo-D. The AUC of ROC analysis was 1.00 (95% CI: 1.00–1.00; *p* = 0.002).

## 4. Discussion

In this large cohort of patients with hypoaldosteronism, we explored whether PAC levels could assist in classifying individuals as Aldo-D or Aldo-R when the classification was based on clinical factors, with diagnostic accuracy assessed separately for each subtype. We found heterogeneous accuracy of PAC levels depended on whether they were assessed during hyperkalemia or hyperreninemia—the two main physiological stimuli of aldosterone release measurable in clinical practice, underscoring several factors that potentially influence results. Consequently, their interpretation is complex.

Based on our ROC analyses, PAC levels did not reliably differentiate hypoaldosteronism due to Aldo-R or Aldo-D when measured during hyperkalemia, nor did they adequately detect Aldo-R when measured during hyperreninemia. However, PAC was significantly informative for identifying Aldo-D in the setting of hyperreninemia, a finding consistent with our multivariable and hierarchical regression analyses. Given these results, and despite the limited ROC performance, we conducted an in-depth evaluation of extreme PAC values—higher (≥200 pg/mL) and lower (≤60 pg/mL) thresholds derived from physiological studies and consistent with those suggested by the Youden Index—in all assessed conditions. Notably, this approach showed that extreme values may be useful for identifying Aldo-R and Aldo-D, as reflected by high specificity and PPV, but they lack the sensitivity required for reliable detection.

An explanation for the overall low diagnostic accuracy of PAC likely lies in the influence of additional factors affecting aldosterone synthesis or action, which were prevalent across all study groups. In fact, the exclusion of cases with CombinedF showed a trend toward improved PAC performance for identifying Aldo-D in the hyperkalemic setting and a significant improvement in the hyperreninemic setting, as well as for Aldo-R under hyperreninemia. However, these findings should be considered exploratory, as this approach substantially reduced the sample size within the analyzed subgroups.

Particular attention should be given to the potential impact of glucocorticoid-induced ACTH suppression, as a substantial rate of patients were receiving glucocorticoid therapy. ACTH is an important stimulator of aldosterone production [35], especially when RAAS-mediated stimulation is impaired [36], such as in patients treated with ACEI or ARBs. The inability to adjust PAC values for ACTH levels represents a limitation that may have influenced our findings.

Altogether, these findings indicate that although PAC could primarily reflect the capacity for aldosterone synthesis, its isolated interpretation has limited utility for accurately distinguishing hypoaldosteronism subtypes since overlapping etiopathogenic factors influencing its values are frequently present in real-world clinical practice. However, extreme PAC values may still help suggest—but not rule out—a particular subtype, underscoring the inherent challenges of applying this classification in routine clinical practice.

### 4.1. Evaluation of PAC Accuracy During Hyperkalemia

The ROC analysis of PAC during hyperkalemia did not reach statistical significance in the primary analysis. However, extreme high and low PAC thresholds showed high specificity for detecting Aldo-R and Aldo-D, respectively, in this scenario.

On the one hand, PAC levels ≥ 168 pg/mL (16.8 ng/dL, ~465 pmol/L)—and especially ≥200 pg/mL (20 ng/dL, ~550 pmol/L)—yielded excellent specificity and PPV (the latter 100%), though with low sensitivity, for Aldo-R. This supports preserved aldosterone production in this subtype, in which a marked PAC rise during hyperkalemia is expected [7]. These results are consistent with the study by Dluhy et al. in 1972 [28], to our knowledge, the only one to define expected aldosterone levels during experimentally induced hyperkalemia, in which ten healthy subjects consistently showed PAC ≥ 200 pg/mL. Thus, values of this magnitude may be interpreted as physiologically expected during hyperkalemia and therefore supportive of Aldo-R in the evaluation of hypoaldosteronism.

On the other hand, PAC values ≤ 63.5 pg/mL (6.3 ng/dL, ~176 pmol/L) and ≤60 pg/mL (6 ng/dL, ~166 pmol/L) showed 90% specificity and ~95% PPV—though similarly low sensitivity—for Aldo-D. Accordingly, such values may help to identify this subtype, although higher values cannot exclude it. Notably, these most accurate thresholds closely matched those derived from physiological studies, suggesting that PAC values below this range could be reasonably considered “low” in the context of hyperkalemia-driven stimulation.

### 4.2. Evaluation of PAC Accuracy During Hyperreninemia

In the absence of universally established cutoffs for hyperreninemia, we defined elevated renin as values within the 4th quartile of our cohort (DRC > 35 pg/mL). This threshold coincided with high levels of renin that have been associated with adverse clinical outcomes in other contexts [25,26,37,38,39] and is consistent with the cutoff used in a previous study evaluating renin-driven aldosterone responses [40]. Therefore, using this upper quartile provides a pragmatic and physiologically grounded framework for interpreting PAC in the context of hyperreninemia and it enabled us to address our study objective.

For Aldo-R in the context of hyperreninemia, ROC analysis yielded an acceptable AUC of 0.732, although reaching only a trend toward statistical significance. PAC ≥ 163 pg/mL (16.3 ng/dL, ~452 pmol/L) showed excellent performance, with specificity and PPV of 100% but low sensitivity (43.8%). PAC ≥ 200 pg/mL had a similar profile, maintaining excellent specificity and PPV, although with a slight reduction in sensitivity (37.5%), as expected.

For Aldo-D, PAC demonstrated very good diagnostic performance, with a significant AUC of 0.917. The highest accuracy was observed at PAC ≤ 210 pg/mL (21 ng/dL, ~582 pmol/L), yielding 85% sensitivity and 100% specificity. However, this analysis was limited by the very small size of the non-Aldo-D group (n = 3), and results should be interpreted cautiously. As expected, PAC ≤ 60 pg/mL preserved specificity but showed lower sensitivity.

As with hyperkalemia, no established PAC thresholds exist for hyperreninemia, and unlike the former, there are no prior physiological studies from which expected aldosterone responses to high renin can be derived. Nonetheless, our results suggest a notable consistency across stimulated PAC cutoffs under both hyperkalemia and hyperreninemia, with values ≥ 160 pg/mL appearing physiologically adequate, and values ≤ 65 pg/mL clearly inadequate. Further dedicated studies are required to confirm these preliminary observations, while these thresholds should not be considered universal diagnostic values.

### 4.3. Additional Insights into the Aldosterone–Potassium–Renin Relationship

A strong correlation between PAC and renin was observed in the overall cohort, particularly among individuals with hyperkalemia. This suggests that, in a substantial proportion of hypoaldosteronism cases, renin is capable of stimulating aldosterone secretion largely independent of [K+] levels. In fact, in our multivariable regression models, renin emerged as the dominant determinant of PAC.

Notably, this correlation within hyperkalemic subgroup remained consistent across all subgroups, except for the non-Aldo-R group, in which no ResFs were identified. This supports the interpretation that, in this subgroup, the underlying mechanism was likely true aldosterone deficiency, whereas in the remaining groups resistance or overlapping mechanisms may have contributed—potentially blunting PAC values and influencing the observed results.

Likewise, a strong correlation between PAC and renin was observed during hyperreninemia, further reinforcing the findings of the regression analysis. However, when evaluating PAC levels, we found only a trend toward higher values among subjects with hyperreninemia than without it. This pattern persisted within the Aldo-D and Aldo-R groups but was no longer evident in their respective counterparts. These findings suggest that once renin levels are markedly elevated, its ability to further modulate PAC is minimally affected by the underlying subtype of hypoaldosteronism. This interpretation is consistent with the regression analysis, in which the independent effect of AldDefF was minimal and that of ResF was null once the predominant influence of renin was evaluated. Additional explanations may include overlapping or coexisting factors that interfere with PAC responses despite renin stimulation, as discussed previously.

This marked relationship between aldosterone and renin should not be interpreted as a limitation; rather, it supports the potential role of PAC in distinguishing Aldo-D and Aldo-R phenotypes by considering that aldosterone production is largely driven by the downstream effect of the underlying mechanism on renin secretion. In Aldo-D, renin levels may be either low or elevated, depending on volume status. In contrast, in Aldo-R, renin is typically elevated. In fact, in our cohort, those with Aldo-R exhibited significantly higher rates of hyperreninemia and renin levels than non-Aldo-R cases. Furthermore, this physiological framework may explain why the independent effect of AldoDefF was weak and that of ResF was null, as the influence of both on aldosterone secretion is likely predominantly mediated through renin.

Overall, these findings underscore the complexity of aldosterone regulation in hypoaldosteronism and reinforce the concept of this condition as a heterogeneous syndrome, in which reduced mineralocorticoid activity represents the central pathogenic axis within a broader physiopathologic spectrum.

### 4.4. Potential Clinical Implications

These findings may have important clinical implications, as they could help clinicians identify hypoaldosteronism subtypes. Based on our results, we think that PAC values above 160 pg/mL, especially above 200 pg/mL, may be suggestive of aldosterone resistance, whereas values ≤ 60 pg/mL may point to aldosterone deficiency when evaluated within the appropriate clinical context similar to our cohort, in the presence of either hyperkalemia or hyperreninemia. However, caution is advised, as intermediate PAC levels do not preclude the involvement of either mechanism in the pathogenesis of hypoaldosteronism, potentially leading to a combined etiopathogenic element.

Aldosterone deficiency and aldosterone resistance may require different therapeutic approaches based on their underlying pathophysiology. The former is most likely to benefit from mineralocorticoid replacement (e.g., fludrocortisone or intravenous hydrocortisone), while the latter may respond better to correction of tubular unresponsiveness (e.g., ensuring adequate fluid and salt delivery or adjusting/discontinuing interfering medications such as cotrimoxazole or MRB) [3,7,8]. Conversely, therapies effective for Aldo-D are unlikely to benefit Aldo-R.

While offering specific treatment strategies for patients with overlapping mechanisms remains speculative, therapies should be individualized. In our experience, decisions are guided primarily by the assumed dominant mechanism, with PAC values interpreted alongside the overall clinical assessment to inform the most appropriate strategy.

Thus, our approach when mixed Aldo-D/Aldo-R mechanism is suspected includes obtaining a detailed history to establish the temporal relationship between potential causal factors and the onset of hypoaldosteronism, as well as assessing volemic status. If no clear causal temporal link is identified, management is mainly volemia-driven. In hypovolemic patients, we tend to initiate oral or intravenous saline together with mineralocorticoid replacement. In euvolemic patients, we typically start a thiazide diuretic while ensuring adequate salt intake, and delay mineralocorticoid replacement for 24–48 h, introducing it only if hyperkalemia persists [41]. Nevertheless, this strategy should not be interpreted as a formal recommendation but rather as an experience-based approach in a complex and understudied clinical scenario. Prospective studies are needed to better define optimal management in these patients.

In summary, the results of this study provide a practical and physiologically grounded framework that—particularly when extreme PAC values are present—may potentially assist clinicians in guiding management, while acknowledging that its therapeutic relevance remains hypothetical and requires prospective validation.

### 4.5. Limitations

Our study has several limitations. First, its retrospective and real-world design reduced the number of cases with PAC measured under hyperkalemia or hyperreninemia, as well as the subtypes evaluated, limiting statistical power. Second, Aldo-D and Aldo-R factors frequently coexist in clinical practice, which may influence aldosterone production and lead to misclassification when PAC values are not extreme. Third, PAC was assessed using RIA, an assay with known variability compared with LC-MS/MS, making absolute thresholds less generalizable [42]. Furthermore, the pre-analytic heterogeneity of PAC and renin measurement may affect the reproducibility and transferability of the proposed thresholds. However, in hypoaldosteronism, volume status, sodium intake, electrolyte disturbances, and RAAS-modifying medications are often the primary pathophysiological drivers, and hormonal values must be interpreted within the patient’s clinical context. Fourth, the absence of established reference ranges for PAC during hyperkalemia or hyperreninemia—as well as the lack of a standardized definition of hyperreninemia—required cohort-based operational definitions. Finally, we focused on diagnostic performance and did not evaluate treatment response or long-term outcomes, nor did we adjust analyses for potential confounders due to the reduction in the sample size and loss of interpretability. Despite these limitations, this study provides the first systematic evaluation of PAC for the classification of hypoaldosteronism and identifies physiologically meaningful thresholds with potential clinical relevance. However, the absence of internal validation introduces a risk of overfitting, and the proposed PAC cutoffs should therefore be considered exploratory. External validation in independent cohorts is required before these thresholds can be confidently applied in clinical practice.

## 5. Conclusions

In conclusion, PAC alone has limited accuracy for distinguishing Aldo-R from Aldo-D, likely due to confounding factors affecting aldosterone production. Nevertheless, PAC values above 160 pg/mL (~443 pmol/L), particularly ≥200 pg/mL (~550 pmol/L), and ≤60 pg/mL (~166 pmol/L) in the presence of hyperkalemia or hyperreninemia may suggest Aldo-R and Aldo-D, respectively. PAC values outside these thresholds should not be interpreted as excluding either subtype, as overlapping mechanisms may be present, and diagnostic decisions should be guided by clinical context. These findings reinforce the need for individualized interpretation of aldosterone and renin in routine clinical practice. Prospective studies are needed to validate these thresholds and clarify their clinical utility.

## Figures and Tables

**Table 1:** Criteria used for diagnosis of hypoaldosteronism.

**A**. Non-fictitious persistent hyperkalemia: -At least 2 laboratory tests with hyperkalemia performed on different days -In addition to: ---Absence of an external potassium load ---Glomerular filtration rate > 30 mL/min/1.73 m^2^
**B.** Hypovolemic hyponatremia with elevated urine sodium (≥30 mmol/L) persisting after diuretic withdrawal, having ruled out: -Bicarbonate administration -Cerebral salt wasting -Proximal tubular disorder
**C.** Absence of hypokalemia
**D.** An adequate therapeutic response: -Improvement of the electrolyte disorder(s) with mineralocorticoid replacement therapy and/or volume and salt repletion with isotonic saline, hypertonic saline, or increased water and salt intake.

Diagnosis required the presence of **A** and/or **B**, together with **C** and **D**.

**Table 2:** Comparison between cases with and without factors related to mineralocorticoid resistance.

	Total (N = 84)	Non-Aldo-R (N = 43)	Aldo-R (N = 41)	*p*
Age, years	77 [65–84]	75 [63–83]	77 [69–84]	0.212
≥65 years	65 (77.4)	30 (69.8)	35 (85.4)	0.088
Female, n (%)	39 (46.4)	26 (60.5)	13 (31.7)	0.008 *
**Comorbidities ^¥^**				
Diabetes mellitus, n (%)	39 (46.4)	20 (46.5)	19 (46.3)	0.988
Hypertension, n (%)	60 (71.4)	29 (67.4)	31 (75.6)	0.426
Chronic kidney disease, n (%)	29 (34.5)	11 (25.6)	18 (43.9)	0.078
Primary adrenal insufficiency, n (%)	4 (5.6)	3 (7.7)	1 (3)	0.620
Hypoalbuminemia, n (%)	28 (33.3)	8 (18.6)	20 (48.8)	0.003 *
Low salt intake, n (%)	40 (47.6)	19 (44.2)	21 (51.2)	0.519
**Concomitant drugs ^¥^**				
Glucocorticotherapy, n (%)	15 (17.9)	1 (2.3)	14 (34.1)	<0.001 *
Heparin, n (%)	24 (28.6)	8 (18.6)	16 (39)	0.038 *
NSAID, n (%)	8 (9.5)	4 (9.3)	4 (9.8)	0.999
β-Blockers, n (%)	14 (16.7)	5 (11.6)	9 (22)	0.250
ACEI/ARB, n (%)	43 (51.2)	24 (55.8)	19 (46.3)	0.385
Loop diuretics, n (%)	17 (20.5)	4 (9.3)	13 (31.7)	0.014 *
Thiazides, n (%)	11 (13.1)	4 (9.3)	7 (17.1)	0.345
**Clinical manifestations**				
Hyperkalemia, n (%) ^¶^	80 (95.2)	39 (90.7)	41 (100)	0.116
Hyponatremia, n (%) ^¶^	70 (83.3)	32 (74.4)	38 (92.7)	0.039 *
Hypovolemic hyponatremia, n (%) ^¶^	54 (64.3)	24 (55.8)	30 (73.2)	0.097
Metabolic acidosis, n (%) ^¶^	32/59 (54.2)	8 (34.8)	24 (66.7)	0.017 *
Hypovolemia, n (%)	49 (58.3)	21 (48.8)	28 (68.3)	0.071
**Biochemical data**				
Aldosterone, pg/mL	90 [41–151]	77 [40–126]	99 [47–248]	0.091
Renin, pg/mL	15 [6–36]	10 [4–18]	27 [8–56]	0.004 *
[K+], mmol/L	5.1 ± 0.5	5 ± 0.5	5.1 ± 0.5	0.341
[Na+], mmol/L	133 ± 6	134 ± 5	132 ± 6	0.054
Serum bicarbonate, mmol/L	23.4 ± 3.4	24.6 ± 2.8	22.6 ± 3.6	0.033 *
Serum creatinine mg/dL	1.1 ± 0.5	1 ± 0.5	1.2 ± 0.6	0.119
TTKG	4 ± 1.3	4 ± 1.1	4.1 ± 1.5	0.597

NSAID: non-steroidal anti-inflammatory drugs; ACEI: angiotensin-converting enzyme inhibitors; ARB: angiotensin 2 receptor blockers; TTKG: trans-tubular potassium gradient. ^¥^ factors related to mineralocorticoid resistance are excluded. ^¶^ represents manifestations found during all episodes of hypoaldosteronism and not only coinciding with aldosterone measurement. * *p* < 0.05.

**Table 3:** Comparison between cases with and without factors related to aldosterone deficit.

	Total (N = 84)	Non-Aldo-D (N = 18)	Aldo-D (N = 66)	*p*
Age, years	77 [65–84]	78 [69–85]	77 [65–83]	0.79
≥65 years	65 (77.4)	14 (77.8)	51 (77.3)	0.999
Female, n (%)	39 (46.4)	11 (61.1)	28 (42.4)	0.159
**Comorbidities ^¥^**				
Diabetes mellitus, n (%)	39 (46.4)	9 (50)	30 (45.5)	0.732
Hypertension, n (%)	60 (71.4)	9 (50)	51 (77.3)	0.023 *
Chronic kidney disease, n (%)	29 (34.5)	3 (16.7)	26 (39.4)	0.096
Obstructive uropathy, n (%)	21 (25)	3 (16.7)	18 (27.3)	0.541
Renal transplant, n (%)	4 (4.8)	0	4 (6.1)	0.573
Urinary tract infection, n (%)	9 (10.7)	1 (5.6)	8 (12.1)	0.676
Hypoalbuminemia, n (%)	28 (33.3)	1 (5.6)	27 (40.9)	0.04 *
Low salt intake, n (%)	40 (47.6)	9 (50)	31 (47)	0.82
**Concomitant drugs ^¥^**				
Glucocorticotherapy, n (%)	19 (22.6)	1 (5.6)	18 (27.3)	0.06
Trimethoprim, n (%)	13 (15.5)	1 (5.6)	12 (18.2)	0.281
Cyclosporine, n (%)	1 (1.2)	0	1 (1.5)	0.999
Tacrolimus, n (%)	4 (4.8)	0	4 (6.1)	0.573
MRB, n (%)	8 (9.5)	2 (11.1)	6 (9.1)	0.678
Loop diuretics, n (%)	17 (20.5)	2 (11.1)	15 (22.7)	0.343
Thiazides, n (%)	11 (13.1)	1 (5.6)	10 (15.2)	0.682
**Clinical manifestations**				
Hyperkalemia, n (%) ^¶^	80 (95.2)	18 (100)	62 (93.9)	0.573
Hyponatremia, n (%) ^¶^	70 (83.3)	11 (61.1)	59 (89.4)	0.004 *
Hypovolemic hyponatremia, n (%) ^¶^	54 (64.3)	6 (33.3)	48 (72.7)	0.002 *
Metabolic acidosis, n (%) ^¶^	32/59 (54.2)	5/13 (38.5)	27/46 (58.7)	0.196
Hypovolemia, n (%)	49 (58.3)	7 (38.9)	42 (63.6)	0.059
Aldosterone, pg/mL	90 [41–151]	90 [68–209]	90 [36–149]	0.79
Renin, pg/mL	15 [6–36]	9 [4–22]	17 [7–45]	0.128
[K+], mmol/L	5.1 ± 0.5	5.1 ± 0.5	5.1 ± 0.5	0.994
[Na+], mmol/L	133 ± 6	135 ± 5	133 ± 6	0.093
Serum bicarbonate, mmol/L	23.4 ± 3.4	24.1 ± 3.8	23.1 ± 3.4	0.453
Serum creatinine mg/dL	1.1 ± 0.5	1.1 ± 0.4	1.1 ± 0.5	0.729
TTKG	4 ± 1.3	3.8 ± 1.2	4.1 ± 1.3	0.525

MRB: mineralocorticoid receptor blocker; TTKG: trans-tubular potassium gradient. ^¥^ factors related to aldosterone deficit are excluded. ^¶^ represents manifestations found during all episodes of hypoaldosteronism and not only coinciding with aldosterone measurement. * *p* < 0.05.

## Data Availability

The data used in this study are available upon reasonable request to the corresponding author and subject to ethical and confidentiality restrictions.
